# Brain evolution in Proboscidea (Mammalia, Afrotheria) across the Cenozoic

**DOI:** 10.1038/s41598-019-45888-4

**Published:** 2019-06-27

**Authors:** Julien Benoit, Lucas J. Legendre, Rodolphe Tabuce, Theodor Obada, Vladislav Mararescul, Paul Manger

**Affiliations:** 1 Evolutionary Studies Institute (ESI), University of the Witwatersrand, Braamfontein, 2050 Johannesburg South Africa; 2 Jackson School of Geosciences, The University of Texas at Austin, 2275 Speedway Stop C9000, Austin, TX United States; 3 Institut des Sciences de L’Evolution de Montpellier, Université Montpellier 2, Place Eugène Batillon, F-34095 Montpellier, cedex 05 Montpellier, France; 4 Academy of Sciences of Moldova, Institute of Zoology, Chişinău, Moldova; 5 School of Anatomical Sciences, University of the Witwatersrand, 7 York Road, Parktown, 2193 Johannesburg South Africa

**Keywords:** Palaeontology, Neuroscience, Palaeontology

## Abstract

As the largest and among the most behaviourally complex extant terrestrial mammals, proboscideans (elephants and their extinct relatives) are iconic representatives of the modern megafauna. The timing of the evolution of large brain size and above average encephalization quotient remains poorly understood due to the paucity of described endocranial casts. Here we created the most complete dataset on proboscidean endocranial capacity and analysed it using phylogenetic comparative methods and ancestral character states reconstruction using maximum likelihood. Our analyses support that, in general, brain size and body mass co-evolved in proboscideans across the Cenozoic; however, this pattern appears disrupted by two instances of specific increases in relative brain size in the late Oligocene and early Miocene. These increases in encephalization quotients seem to correspond to intervals of important climatic, environmental and faunal changes in Africa that may have positively selected for larger brain size or body mass.

## Introduction

In mammals, brains larger than 700 g have evolved infrequently, being found primarily in extant humans, cetaceans and elephants^1^. The temporal/evolutionary history of human brain size and shape has been studied in detail^2–5^, with the 700 g barrier on brain size evolution being surpassed with the appearance of *Homo ergaster*/*erectus*, approximately 1.8 million years ago^1^. The suborder Cetacea has many species that also exhibit large brains, both absolute and relative to body mass^6,7^. It has been established that large cetacean brains emerged with the evolution of the Neoceti approximately 32 million years ago^8,9^. In contrast to humans and cetaceans, the temporal and evolutionary history of brain size in Proboscidea is poorly understood^1,10^.

Elephants are the largest and among the most behaviourally complex terrestrial mammals. They are known for their extensive long-term memory, problem-solving abilities, behavioural adaptability, their ability to recognize themselves in a mirror, to manipulate their environment, and to manufacture tools with their trunk^11,12^. On the one hand, given that the metabolic cost of brain tissue is high^13^, and following the assumption that natural selection would not maintain a costly organ that brings no benefit, the presence of a large brain in elephants suggests there is some adaptive value, potentially related to their cognitive capacities. It has been proposed that evolutionary selective pressures requiring increased intelligence (e.g. cognitive and sensory processing, memory, behavioural flexibility) to cope with a variety of environmental variables (e.g. sociality, gregariousness, diet, habitat) has driven the evolution of large brains in both Primates and ‘ungulates’^14–17^. This is based on the retrospective assignation of implied cognitive needs centred on the apparent behavioural flexibility and intelligence of extant species. Similarly, this retrospectively applied cognitive explanatory paradigm has been argued to apply to the evolution of a large brain in cetaceans^18–20^, and elephants^10,12^; however, alternative explanations of brain size evolution have been forwarded, focussing on features such as the structural laws of form linking body and brain mass over evolutionary time^1,7,21,22^. Under this assumption, the elephantine brain is a typical mammalian brain that appears “to scale consistently with the scaling laws governing brain and body mass relationships across mammals”^1^. In parallel, the assumption that complex cognition requires an increase in brain size has been heavily criticized^1,21,23,24^. Therefore whether the evolution of a large brain is an adaptive trait (e.g.)^10^, or simply accompanied the increase in body mass in proboscideans (e.g.)^1^, remains an open question.

While proboscidean evolution is well documented and understood^25–30^, studies of the brains and endocranial casts of extinct proboscidean genera are limited^10,12,31–35^. These studies have indicated that brain size, both in absolute and relative terms, appears to have increased dramatically in Elephantiformes, sometime between the Oligocene and Pliocene, a poorly constrained interval of more than 30 million years^10^. Thus, the timing and rate of changes in brain size, and the taxa involved in the evolution of a large brain during proboscidean evolution remains unclear. Building on the database of Benoit^10^, by adding data from three key taxa, *Zygolophodon* (*Mammut*) *borsoni*, *Palaeomastodon beadnelli* and *Stegodon insignis*, we apply statistical and phylogenetic comparative methodology to changes in brain size in proboscideans throughout their evolutionary history. To address whether a large brain in proboscideans evolved as an adaptation or as a consequence of increased body mass, we examined the temporal scaling relationships of brain with body size and with associated environmental changes that have occurred during proboscidean evolutionary history.

## Methods

### Data collection

Data analysed (endocranial volume and body mass) are summarized in Table 1. Data for extant species of elephants are from Benoit *et al*.^36^ (note that this dataset contained two duplicated lines that are removed here) and those for extinct species are from Benoit^10^. The body masses of some extinct taxa were updated using the recent estimates of Larramendi and Palombo^32^ and Larramendi^37^ (see Table 1).

**Table 1:** Endocast, calculated brain size and Manger’s EQ^7^ for the proboscideans used in the analyses.

	Endocast volume (cm^3^)	Brain size (g)	Body mass (g)	EQ Manger
*Elephas maximus*	4550^b^	4181	2267430^b^	1,83
*Elephas maximus*	5220^b^	4798	3216000^b^	1,62
*Elephas maximus*	5000^b^	4595	3450400^b^	1,48
*Elephas maximus*	6075^b^	5584	3190098^b^	1,90
*Loxodonta africana*	5712^b^	5250	6654000^b^	1,05
*Loxodonta africana*	9000^b^	8274	4380000^b^	2,23
*Loxodonta africana*	4000^b^	3676	5174400^b^	0,88
*Loxodonta africana*	4050^b^	3722	1793300^b^	1,93
*Loxodonta africana*	4420^b^	4062	3505000^b^	1,29
*Loxodonta africana*	5300^b^	4871	5550000^b^	1,11
*Loxodonta africana*	4480^b^	4117	2750000^b^	1,56
*Loxodonta africana*	4210^b^	3869	4000000^b^	1,12
*Loxodonta africana*	4100^b^	3768	2160000^b^	1,70
*Loxodonta africana*	4000^b^	3676	2537000^b^	1,48
*Palaeoloxodon falconeri*	1800^a^	1652	168000^4^	4,81
*Palaeoloxodon antiquus*	5446^a^	5005	3649880^a^	1,54
*Palaeoloxodon antiquus*	9000^a^	8274	11000000^c^	1,14
*Mammuthus primigenius*	4687^a^	4307	6000000^d^	0,92
*Mammuthus meridionalis*	5828^a^	5357	11000000^d^	0,74
*Mammuthus columbi*	6232^a^	5728	9800000^d^	0,86
*Mammut americanum*	3862^a^	3549	6384056^a^	0,73
*Mammut americanum*	4630^a^	4255	8000000^d^	0,74
*Stegodon insignis*	3838	3527	2000000^d^	1,69
*Zygolophodon borsoni*	5133	4718	16000000^d^	0,50
*Palaeomastodon beadnelli*	771	706	2500000^d^	0,29
*Moeritherium lyonsi*	240^a^	218	810000^d^	0,20
*Prorastomus sirenoides*	87^b^	90	98156^b^	0,39
*Seggeurius amourensis*	5^b^	5	2932^b^	0,29

The sirenian *Prorastomus* and the hyracoid *Seggeurius* were added for comparative purposes but were not included in the analyses. Sources of data: ^a^ ^10^; ^b^ ^36^;^c^ ^34^;^d^ ^37^.

This dataset was also updated with the addition of endocranial volume and body mass of three taxa: *Zygolophodon* (*Mammut*) *borsoni* (MCFFM-CLB-1), from the Pliocene (Mammal Neogene zone 15) of Otman Hill (Moldova)^38^, *Stegodon insignis* (MNHN-A952) from an unknown Miocene locality in the Himalayas^39^, and *Palaeomastodon beadnelli* (NHMUK PV M 8464) from the early Oligocene of the Fayum deposits (Egypt)^40^. As one of the largest proboscideans that existed^37^, *Z. borsoni* is important for discussing the effect of body mass on the evolution of brain mass in proboscideans. Stegodontids may be the possible outgroup of modern elephantids, and *Palaeomastodon beadnelli* is the basal-most known elephantiform and the closest relative of the clade Elephantimorpha in the dataset^26,28–30,41^. For this study, the braincase of *P. beadnelli* (NHMUK PV M 8464), which is partially exposed^40^, was carefully examined and drawn by a palaeo-artist (Sophie Vrard) in dorsal and ventral views under the supervision of J.B. (Supplementary Fig. 1). The endocranial volume was estimated to 771 cm^3^ using double-graphic integration on the drawings^31^. Radinsky (ref.^42^, p. 84) performed comparisons with the water displacement technique that validated the accuracy of double graphic integration and suggested that this method should be preferred in case of incomplete preservation or when direct measurement is impossible, as is the case here with NHMUK PV M 8464. As originally described, specimen MNHN-A952 is the cast of the right side of the braincase of a *Stegodon insignis* skull assembled with the left endocast of a modern elephant to enable Gervais^39^ to reconstruct a complete endocranial cast (Supplementary Fig. 2). The endocranial size of *Zygolophodon* (*Mammut*) *borsoni* is based on a latex endocranial cast of specimen MCFFM-CLB-1 (Fossil Fauna Complex Museum of Moldova, Chisinau) (Supplementary Fig. 3). To measure its volume, the endocast was digitized using photogrammetry AgiSoft PhotoScan Professional Version 1.3.2), a method that has proven as accurate as CT scanning^43^ and sometimes more accurate than laser-scanning^44,45^. The volume of the resulting mesh was then measured using Avizo (FEI VSG, Hillsboro OR, USA). The resulting volume is 5133 cm^3^ (the *Zygolophodon* endocast was also measured at 4910 cm^3^ using water displacement, but the digital measure was preferred as it less prone to errors)^2^. To measure the volume of the endocast of *Stegodon insignis*, the complete endocast was digitized using photogrammetry, its right half isolated digitally, and the volume of the resulting mesh was measured using the same software programs. The resulting volume, 1919 cm^3^, was then multiplied by two to obtain the volume of a complete endocast. Body masses for these three taxa are from Larramendi^37^. The encephalization quotient (EQ) used is that of Manger^7^, which is very similar to Eisenberg’s EQ^46^, although it excludes outliers such as cetaceans and primates. Brain volumes used to calculate EQ are based on endocranial volumes corrected for meningeal thickness using Benoit’s^10^ method. This is a necessary step as the thick meninges in proboscideans occupy some 14% of the endocranial cavity with non-neural tissue on average^10^. The resulting brain volumes were then multiplied by 1.036, which represents the average specific gravity of brain tissue^32,47^.

### Ancestral character state reconstruction

In order to analyse temporal patterns of endocranial volume, body mass and EQ changes across proboscidean phylogenetic history, an ancestral character state reconstruction using maximum likelihood (ACSRML) was performed using the function fastAnc in the R package ‘phytools’^48^ (excluding the sirenian *Prorastomus* and the hyracoid *Seggeurius*). The phylogeny used for the ACSRML was assembled following the current consensus^28–30,41^ (Supplementary Fig. 4). The resulting dates are consistent with the recently published palaeogenomic study by Palkopoulou *et al*.^49^. There have been many discrepancies concerning the phylogenetic placement of the genus *Palaeoloxodon*. Palaeogenomic studies have consistently placed *P. antiquus* within the African elephant genus *Loxodonta*^49,50^, whereas studies of the mitochondrial DNA have suggested that *P. falconeri* belongs to the Asian elephant genus *Elephas*^51,52^. Given the morphological support for the validity of the genus *Palaeoloxodon*^25,53,54^ and the burden of evidence in favour of a direct ancestor to descendant relationship between *P. antiquus* and *P. falconeri* respectively^29,55,56^, we preferred to remain conservative and preserved the monophyly of the genus *Palaeoloxodon*. Nevertheless, the branch leading to *Palaeoloxodon* is in an unresolved position within Elephantida in order to reflect these phylogenetic inconsistencies (Supplementary Fig. 4). Divergence ages and branch lengths were determined based on published literature^29,57^.

### Phylogenetic regressions

In order to test for the presence of a functional relationship between brain mass and body mass, linear regressions were performed on our datasets (excluding the sirenian *Prorastomus* and the hyracoid *Seggeurius*). In such statistical analyses, there are several assumptions made about the data, such as normality, homoscedasticity and independence of observations. When comparing traits of different species, independence is considered potentially violated because of the evolutionary relationships of species to each other, and thus phylogeny needs to be considered as part of the analysis^58^. All analyses were performed in R v. 3.5.3 (R Core Team, 2019).

Phylogenetic generalized least squares (PGLS)^59^ are a very well-known and frequently used phylogenetic comparative method (see Garamszegi^60^, for an extensive review), designed to consider the influence of evolutionary relationships between species (i.e., phylogenetic signal *sensu* Blomberg and Garland)^61^ in a generalized linear model, using a regression of one or more continuous predictors over a response variable. After a log-conversion to better approximate the allometric relationship between variables of interest^62,63^, endocranial volume was regressed on body mass using a calibrated phylogeny of Proboscidea in the dataset (Supplementary Fig. 4; Supplementary Material 5). Since the evolutionary model followed by this regression is not known, the analysis was performed several times using different evolutionary models, and the goodness of fit of each model determined from their AICc score, i.e., the Akaike Information Criterion corrected for finite sample size^64^. Using the R package ‘AICcmodavg’^65^, AICc scores were compared for five different evolutionary models: Brownian motion, i.e., a purely neutral model of evolution^66^; Ornstein-Uhlenbeck, i.e., a model with selective optima along the branches^67^; lambda model, i.e. modified Brownian motion model with all branch lengths weighted using Pagel’s lambda, a common estimator of phylogenetic signal^68^, here estimated from the dataset through maximum likelihood; Early Burst, i.e., model with high initial evolutionary rates that decelerate through time after a cladogenesis event^69^; and Ordinary Least Squares (i.e simple, non-phylogenetic least squares regression). The model with the lowest AICc score was found to be the lambda model (AICc = −18.2, vs a range of −6.8–10.11 for other evolutionary models), with λ = 0.90, indicating a strong phylogenetic signal in the regression. Thus, the lambda model was selected to perform further analyzes on the dataset. PGLS were performed using the function gls in the R package ‘nlme’^70^, and the phylogenetic correlation structure corPagel (for the lambda model) specified in the package ‘ape’^71^.

**Figure 1:**
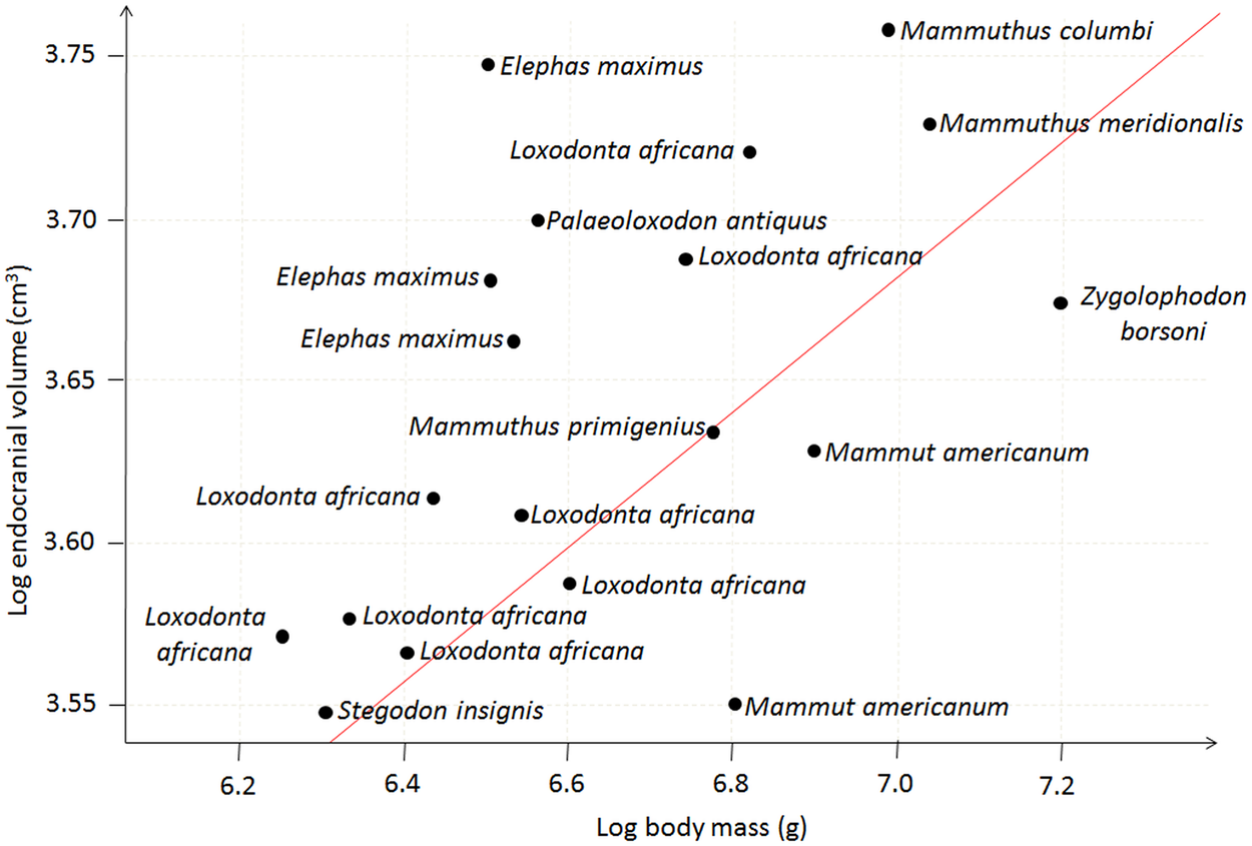
Regressions of log brain mass over log body corrected for phylogeny excluding outliers.

Normality and homoscedasticity of the residuals in the model were assessed using a Lilliefors test in the package ‘nortest’^72^, as well as graphically checked through plotting against corresponding fitted values, to conform to the parametric assumptions of PGLS^73^. Residuals were found to be non-normal, and seven outliers were graphically detected and removed from the dataset (Supplementary Material 6). These outliers consist of *Moeritherium lyonsi*, *Palaeomastodon beadnelli*, *Palaeoloxodon falconeri*, one specimen of *Palaeoloxodon antiquus* as well as one specimen of *Elephas maximus* and two of *Loxondonta africana*. After pruning the corresponding branches in the tree, regression analyses were performed again, and the new residuals were found to be normal and homoscedastic (Fig. 1), allowing for a straightforward interpretation of the new results.

## Results

**Figure 2:**
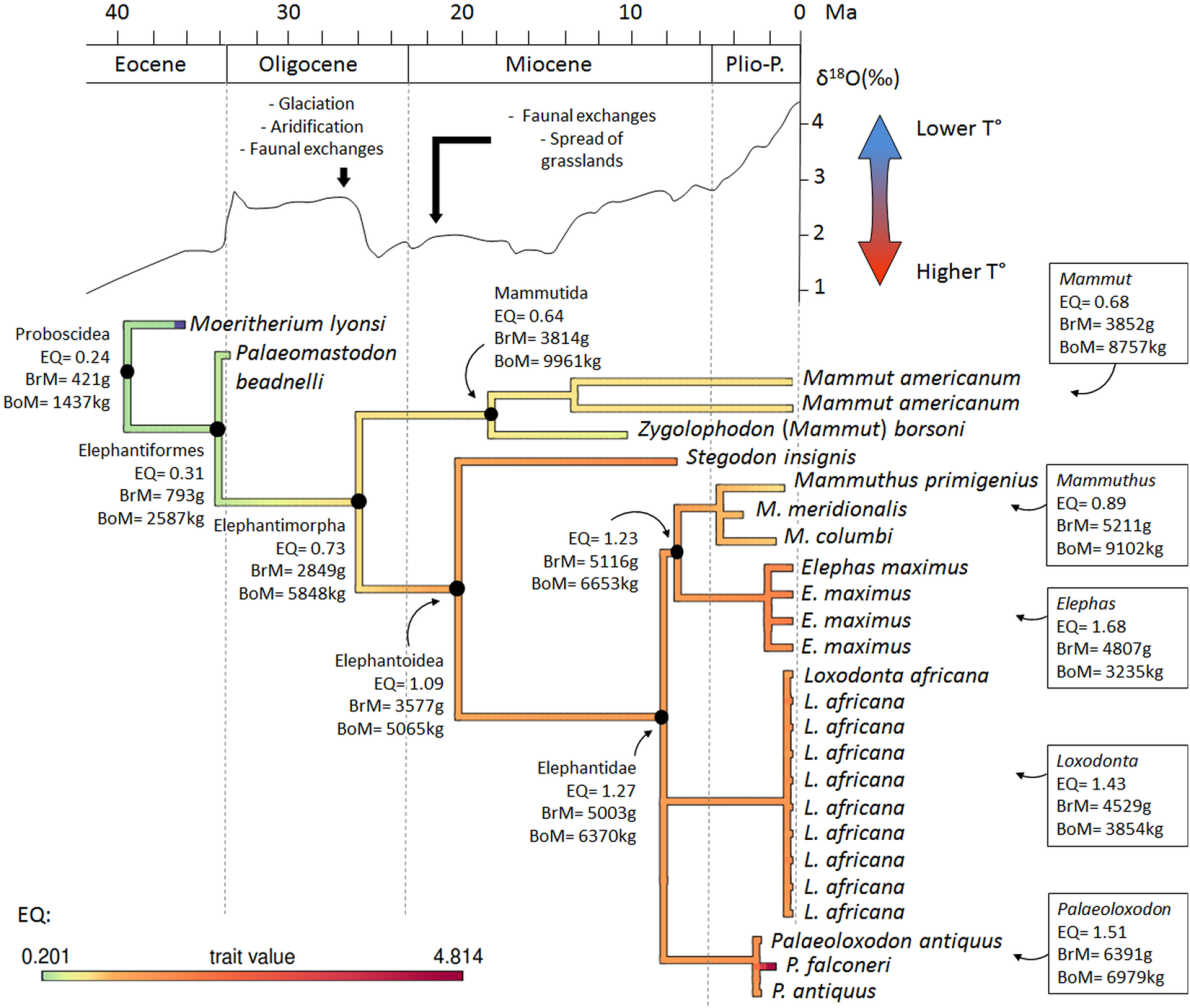
Results of the Ancestral Character State Reconstruction using Maximum Likelihood. Geological time scale and δ18O curve after Zachos *et al*.^84^. Abbreviations: BoM, body mass; BrM, brain mass; EQ, encephalization quotient.

According to the ACSRML, which was performed on the whole dataset (Fig. 2), the EQ in the last common ancestor (LCA) of *Moeritherium* and Elephantiformes is 0.24. It increases slightly in the next node to reach 0.3 in the LCA of Elephantiformes (*Palaeomastodon* and more derived proboscideans). The reconstructed brain and body masses at this node are about 1.8 times larger than in the previous node, while the EQ remains similar, which indicates that during the Paleogene, increases in brain mass were proportional to increases in body mass in basal proboscideans (Fig. 2). The reconstructed EQ doubles in the node corresponding to the common ancestor of Elephantimorpha that lived some 26 Ma ago in the late Oligocene, where it reaches a value of 0.73. At this node, the ACSRML indicates that body mass has increased by 2.3 times while brain mass has increased by 3.6 times compared to the previous node (Fig. 2). The reconstructed EQ then decreases slightly in the lineage leading to Mammutida and *Mammut* (0.64 and 0.68 respectively) in which the reconstructed body mass exceeds 8 tons while brain mass gains only 1 kg (Fig. 2). In contrast, the EQ is nearly doubled again in the LCA of the Elephantoidea that lived some 20 Ma ago in the Early Miocene, to reach 1.09. At this node, the reconstructed body mass decreases slightly while brain mass is reconstructed as being about 1.2 times larger than in the previous node (Fig. 2). Reconstructed ancestral EQs, and brain and body masses then remain relatively stable across Elephantoidea and Elephantidae phylogeny (Fig. 2). Reconstructed ancestral body masses vary between about 4 and 7 tons, while brain masses vary between 4.5 and 6.4 kg in Elephantidae (Fig. 2). Reconstructed variations in the EQ nevertheless appear mostly due to variations in body mass. In the *Mammuthus* clade, the elevated reconstructed body mass (9.1 tons) coincides with a decrease in the reconstructed EQ compared to the ancestral value in the LCA of Elephantoidea to 0.89 (Fig. 2). In contrast, the increase in EQ to 1.68 in the *Elephas* clade is accompanied by a decrease in ancestral body mass to 3.2 tons (Fig. 2). The ancestral value for the *Palaeoloxodon* clade is surprisingly close to that for the LCA of Elephantidae (1.51).

The EQ varies around 1.47 ± 0.76 among all Elephantimorpha except in two taxa that display extreme EQ values. On one end of the spectrum is the small bodied and highly encephalized dwarf elephant of Sicily, *Palaeoloxodon falconeri*, which displays an EQ of 4.81 (Table 1). This species is well studied and its large EQ has been extensively discussed in the literature^10,32,34^. Its body size is between one tenth and one hundredth that of its close relative, *Palaeoloxodon antiquus*, whereas its endocast volume is about one half to one quarter that of *P. antiquus*^10,32,34^ (Table 1). On the opposite end of the spectrum is *Zygolophodon (Mammut) borsoni* which, with an estimated body mass of 16 tons^37^, is the heaviest species in the dataset. The *Zygolophodon* EQ of 0.50 also makes it the least encephalized of all Elephantimorpha, although the clade to which it belongs, the Mammutida, ancestrally display a low EQ value (0.64) similar to that reconstructed for the ancestral Elephantimorpha (Fig. 2).

The regression of brain mass over body mass corrected for phylogeny and excluding outliers (Fig. 1) shows that the relationship between the two variables is significant (p-value = 0.0004), but their correlation is relatively weak (R² = 0.50) and the slope of the regression line is low (0.20). This indicates that brain and body mass are significantly correlated, but this correlation and the effect of the brain and body mass on each other are moderate.

When outliers are included (Supplementary Material 6), the correlation between the two variables is slightly higher (R² = 0.61) and remains significant (P-value < 0.0001); however, since the assumptions of normality and homoscedasticity of the sample are not met for this analysis, this result is of dubious reliability^73^.

## Discussion

The oldest known endocranial cast of a proboscidean belongs to *Moeritherium lyonsi* from the late Eocene Qasr el Sagha Formation of the Fayum, Egypt^40^. The EQ of *Moeritherium* equals 0.2 (Table 1), which is an order of magnitude smaller than in any Elephantimorpha (Table 1). A comparably low EQ value is also found in *Palaeomastodon* (EQ = 0.29), which was contemporaneous with *Moeritherium*, and in the paenungulates *Seggeurius* (EQ = 0.29) and *Prorastomus* (EQ = 0.39), the basal-most hyracoid and sirenian respectively, and the closest relatives of proboscideans that lived during the early Eocene (~50 Ma)^36^ (Table 1). As a consequence, it is not surprising that a rather small EQ is reconstructed as primitive for Proboscidea and Elephantiformes by the ancestral character state analysis (Fig. 2). The relatively small brain cavity of *Phosphatherium*, a stem proboscidean from the early Eocene (−55 Ma)^74^ supports this assumption.

The analysis also suggests that brain mass evolved proportionally to body size in non-elephantimorph proboscideans (‘Plesielephantiformes’) and among more derived elephantids (Fig. 2), which is supported by the significant correlation between brain and body mass in the dataset (p-value = 0.0004). This would support the idea that brain mass evolved as a ‘simple passenger’ of body mass in these two groups, as hypothesized by Manger^1^. Given the relationship between brain and body mass variation, it can be predicted that non-elephantimorph taxa with body masses already approaching or exceeding two tons, such as *Barytherium* and the Deinotheriidae^27,34,75,76^ would have had an absolutely larger brain than the basal most proboscideans. This assumption might be addressed in the future, when more data on the endocasts of *Barytherium* and the Deinotheriidae becomes available.

In contrast, the ACSRML suggests that brain mass increases much faster than body mass in Elephantimorpha, resulting in two pulses of increase in the EQ: (i) the EQ doubles in the LCA of Elephantimorpha and (ii) it almost doubles again in the LCA of Elephantoidea (Fig. 2). Subsequently, the EQ essentially remains the same among Elephantoidea, including modern proboscideans (Fig. 2). According to the ACSRML (Fig. 2), brain mass began increasing in the basal-most elephantimorphs, the Mammutida (*M. americanum* and *Z. borsoni*), where the EQ is about twice that of *Moeritherium* and *Palaeomastodon* (Table 1). A brain mass equivalent to that of most modern elephants is achieved in the LCA of the Elephantoidea (Fig. 2). These two pulses suggest that, at least in the LCA of Elephantimorpha and Elephantoidea, increases in brain mass were decoupled from increases in body mass, and that an increase in EQ was positively selected for.

What could have driven such increases in EQ? According to both the fossil record and molecular studies, the LCA of Elephantimorpha lived some 26 million years ago in the late Oligocene^28,29,57,77^, and the LCA of the Elephantoidea lived around 20 million years ago in the early Miocene^28,29^. These time points are contemporaneous with two periods of increased aridity on the African continent, accompanied by glaciations, arrival of invasive faunal elements from Eurasia, dispersal of proboscideans out of Africa and the spread of C4 plants^28,78–84^ (Fig. 2). Gregariousness^17,85^, greater behavioural flexibility^86^ and the ability to produce complex long distance, infrasonic calls^12,25,87,88^ have been documented in Miocene elephantiforms and may have played a role in increasing the fitness of relatively larger brained individuals in the context of ecological changes^10,14,15^. An enhanced long term memory may have facilitated the recalling of the location of distant water holes during increasingly frequent droughts^11^, though evidence from the fossil record is lacking^10^. A shift in proboscidean diets (as a result of the advent of the conveyor-belt type of dental replacement and increasing hypsodonty)^77,86^ seems an unlikely driver for the evolution of a higher EQ because (i) proboscideans remained primarily browsers until elephantids became grazers some 7 million years ago^86,89,90^, and (ii) no undisputable correlation is evident between diet and brain size in ‘ungulates’^14,15^.

Alternatively, given that brain and body size variations appear significantly correlated in proboscideans (Fig. 1), it is possible that body mass, not brain mass, could have been the main trait under selective pressure during these two time intervals. Positive selection for larger body size could have provided early elephantimorphs with protection against the novel invasive predators and competitors from Eurasia, and large herbivores are known to be less sensitive to droughts and increased seasonality as they can store more fat and water, and can subsist on lower quality food (e.g. grasslands) due to their larger digestive tract and lower metabolic rate^75,91,92^. In this respect, it is noteworthy that other ‘ungulate’ taxa contemporaneously show an overall increase of their brain and body mass in the early Neogene^1,93,94^. This would make the increase in EQ merely a correlate of the increase of body mass, as already hypothesized by Manger *et al*.^1^. However, brain mass increases faster than body mass in this portion of the tree, and the latter even decreases slightly in the LCA of Elephantoidea compared to the LCA of Elephantimorpha (Fig. 2), which does not match what would be expected under the assumption that body mass was the main driver of EQ increase.

## Conclusion

Overall, there does not seem to be one single hypothesis accounting for the complete evolution of the brain in proboscideans. Part of the evolution of a larger absolute brain mass may be explained by the co-evolution of brain and body mass (in ‘plesielephantiforms’ and elephantids) and part of this process seems to better explained by potential selective pressures (in the LCAs of Elephantoidea and Elephantimorpha), perhaps as a result of climatic and environmental changes in Africa, as well as with dispersal events. Despite this, brain size evolution remains significantly correlated to body size in proboscideans, which cautions against an explanatory scenario involving intelligence alone as the main focus of natural selection. The precise timing of the two identified pulsed increases in relative brain size remains poorly temporally constrained due to the small sample size that makes it difficult to obtain reasonable phylogenetic resolution. Considerable effort will have to be put into collecting data for key taxa that will fill the temporal voids in the current data set. The inclusion of Barytheriidae and Deinotheriidae - as some of the closest relatives to Elephantiformes and the earliest large proboscideans- and investigation of whether they evolved a larger brain independently of other ‘Plesielephantiformes’ will prove crucial in this respect^1^.

## Supplementary information

Supplementary materials

## Data Availability

All the data necessary to reproduce this research are provided with the manuscript.
