# Supplementary material for: Brain evolution in Proboscidea (Mammalia, Afrotheria) across the Cenozoic
Source: Sci Rep. 2019 Jun 27;9:9323. doi: 10.1038/s41598-019-45888-4 (PMC6597534; doi:10.1038/s41598-019-45888-4)
Supplement: Supplementary file 1 — Supplementary materials [file 41598_2019_45888_MOESM1_ESM.pdf]

Brain evolution in Proboscidea (Mammalia, Afrotheria) across the Cenozoic

Julien Benoit[1*], Lucas J. Legendre[2], Rodolphe Tabuce[3], Theodor Obada[4], Vladislav Mararescul[4], Paul Manger[5]

[1]Evolutionary Studies Institute (ESI), University of the Witwatersrand, Braamfontein, 2050, Johannesburg, South Africa

[2]Jackson School of Geosciences, The University of Texas at Austin, 2275 Speedway Stop C9000, Austin, TX, United States

[3]Institut des Sciences de L'Evolution de Montpellier, Université Montpellier 2, Place Eugène Batillon, F-34095 Montpellier cedex 05, Montpellier, France

[4]Academy of Sciences of Moldova, Institute of Zoology, Chişinău, Moldova

[5]School of Anatomical Sciences, University of the Witwatersrand, 7 York Road, Parktown, 2193, Johannesburg, South Africa

Correspondance to: Julien.benoit@wits.ac.za

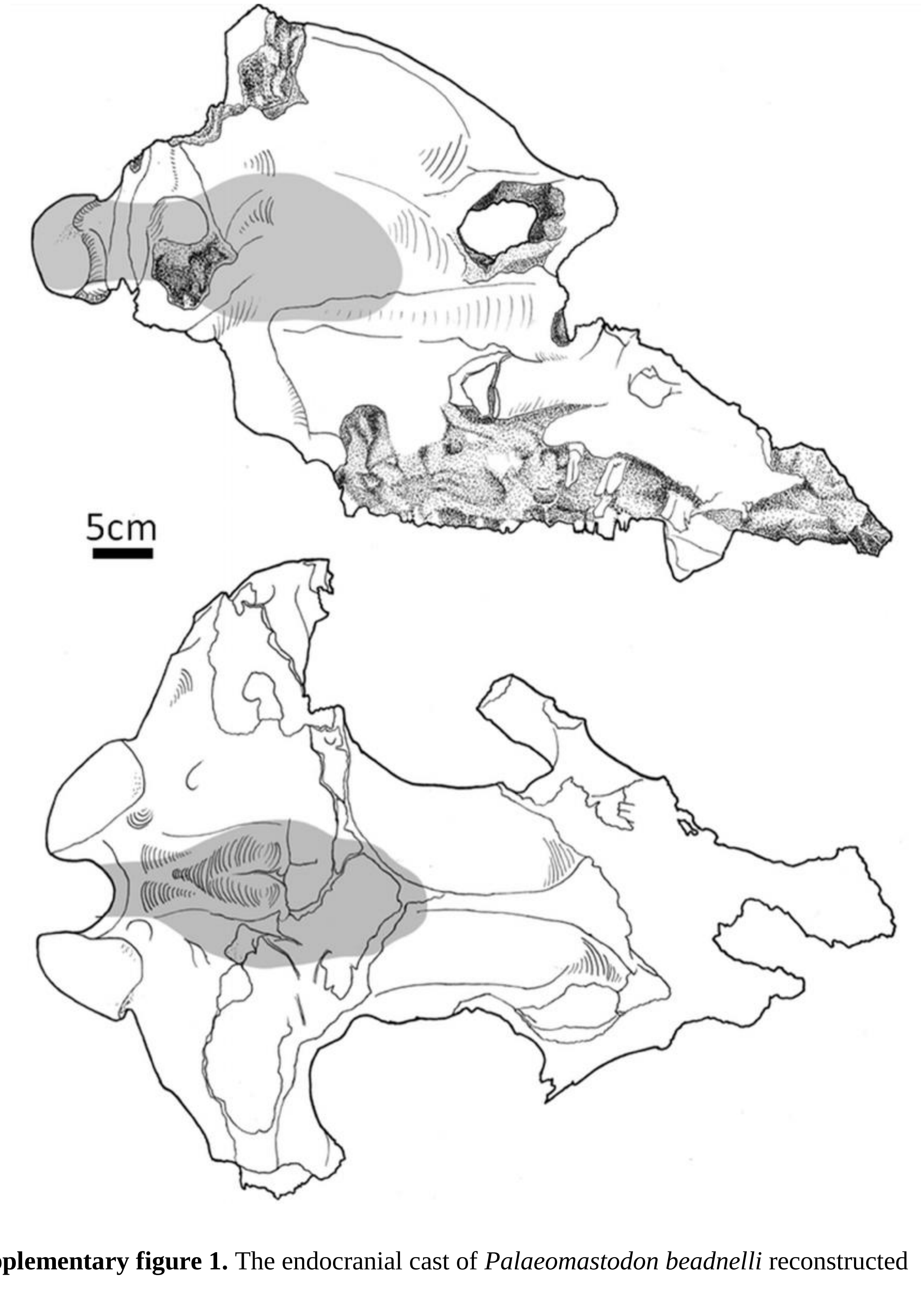

Supplementary figure 1. The endocranial cast of *Palaeomastodon beadnelli* reconstructed inside the transparent skull (NHMUK 8464). Top, lateral view; bottom: dorsal view. Artwork by Sophie Vrard.

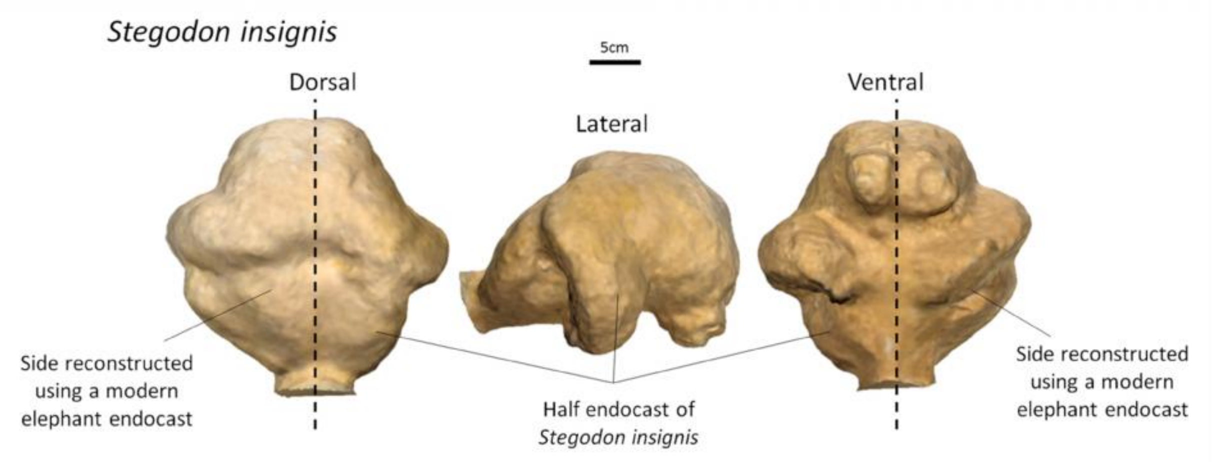

Supplementary figure 2. The digitized endocranial cast of *Stegodon insignis* (MNHN-A952).

Left, dorsal view; middle: lateral view; right, ventral view.

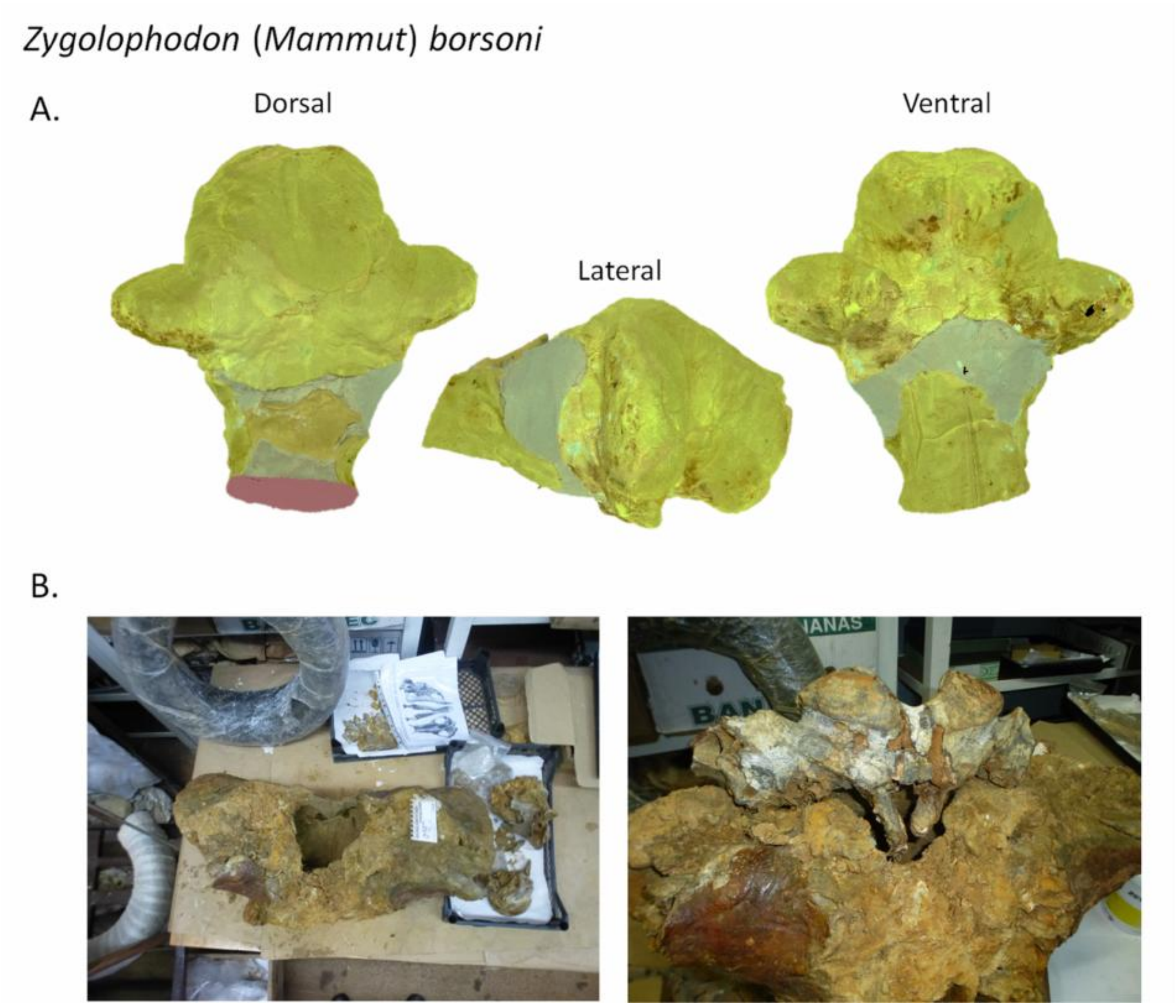

Supplementary figure 3. The endocranial cast of *Zygolophodon* (*Mammut*) *borsoni* (MCFFM-CLB-1). A. The digitized endocast. Left, dorsal view; middle: lateral view; right, ventral view. B. Photos of the actual specimen showing the prepared braincase in caudal view (left) with the occipital condyles removed and in ventral view (right) with the occipital condyles in place.

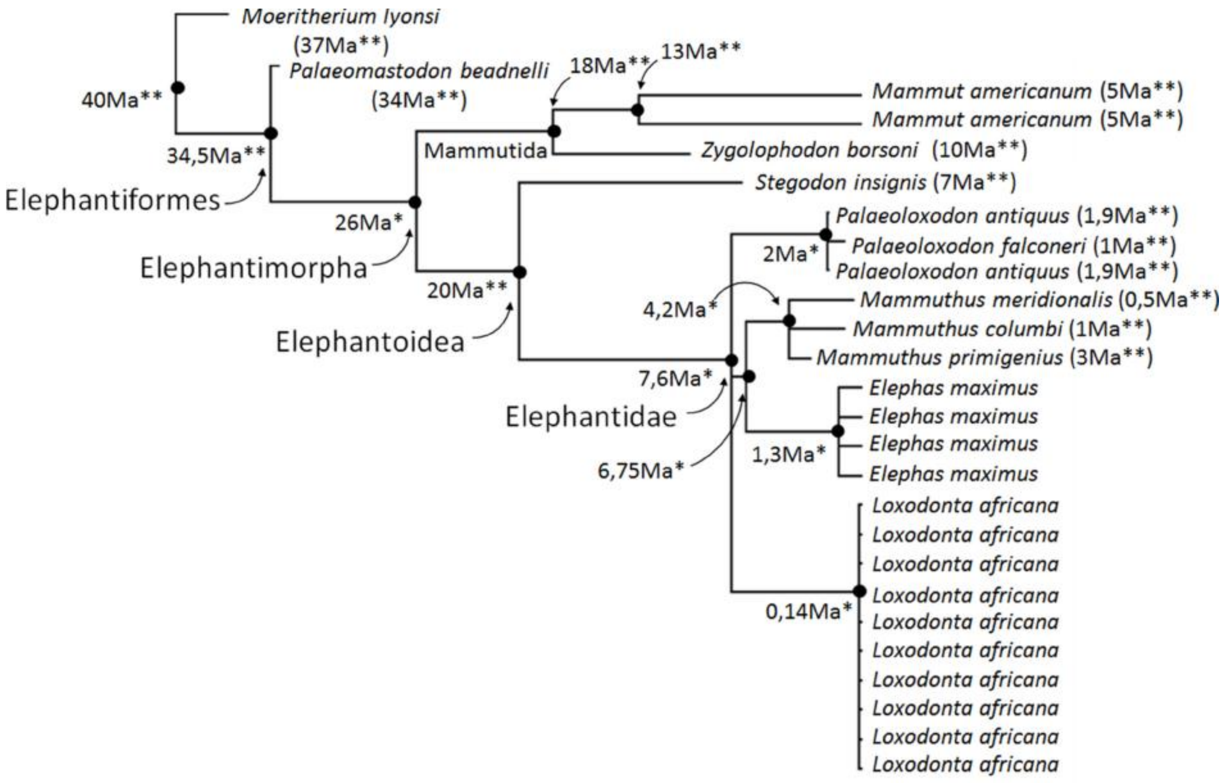

Supplementary figure 4. The phylogeny and divergence ages used for the Ancestral Character State Reconstruction using Maximum Likelihod (Source of the tree: see material and methods). Divergence ages and earliest occurence in the fossil record in million years (Ma) from: *, ref [24]; **, ref [41].

Supplementary material 5. Script from the nexus file containing the dataset and time calibrated phylogenetic tree used for this work.

```
#NEXUS
[written Fri Aug 24 07:41:49 CAT 2018 by Mesquite  version 2.75 (build 564) at User/146.141.25.99]

BEGIN TAXA;
	TITLE Taxa;
	DIMENSIONS NTAX=26;
	TAXLABELS
		Elephas_maximus_1 Elephas_maximus_2 Elephas_maximus_3 Elephas_maximus_4 Loxodonta_africana_1 Loxodonta_africana_2 Loxodonta_africana_3 Loxodonta_africana_4 Loxodonta_africana_5 Loxodonta_africana_7 Loxodonta_africana_8 Loxodonta_africana_9 Loxodonta_africana_10 Loxodonta_africana_11 Palaeoloxodon_falconeri Palaeoloxodon_antiquus_1 Palaeoloxodon_antiquus_2 Mammuthus_primigenius Mammuthus_meridionalis Mammuthus_columbi Mammut_americanum_1 Mammut_americanum_2 Zygolophodon_borsoni Palaeomastodon_beadnelli Moeritherium_lyonsi Stegodon_insignis
	;
```

END;

BEGIN TREES;

Title Afrotheria_tree_block;

LINK Taxa = Taxa;

TRANSLATE

1 Elephas_maximus_1,

2 Elephas_maximus_2,

3 Elephas_maximus_3,

4 Elephas_maximus_4,

5 Loxodonta_africana_1,

6 Loxodonta_africana_2,

7 Loxodonta_africana_3,

8 Loxodonta_africana_4,

9 Loxodonta_africana_5,

10 Loxodonta_africana_7,

11 Loxodonta_africana_8,

12 Loxodonta_africana_9,

13 Loxodonta_africana_10,

14 Loxodonta_africana_11,

15 Palaeoloxodon_falconeri,

16 Palaeoloxodon_antiquus_1,

17 Palaeoloxodon_antiquus_2,

18 Mammuthus_primigenius,

19 Mammuthus_meridionalis,

20 Mammuthus_columbi,

21 Mammut_americanum_1,

22 Mammut_americanum_2,

23 Zygolophodon_borsoni,

24 Palaeomastodon_beadnelli,

25 Moeritherium_lyonsi,

26 Stegodon_insignis;

TREE 'Afrotheria++++++' =
(((((((1:1.3,2:1.3,3:1.3,4:1.3):5.45,(20:3.2,18:3.7,19:1.2[%selected = on
]):2.55):0.85,(5:0.14,6:0.14,7:0.14,8:0.14,9:0.14,10:0.14,11:0.14,12:0.14,13:0.14,14:0.14):7.46,(
15:1.0,17:0.1,16:0.1):5.6):12.4,26:13.0):6.0,(23:8.0,(21:13.0,22:13.0):5.0):8.0):8.5,24:0.5):5.5,25
:3.0)[%] [%] [% setBetweenBits = selected];

ID 01615222172334;

```
END;

Begin MESQUITE;
		MESQUITESCRIPTVERSION 2;
		TITLE AUTO;
		tell ProjectCoordinator;
		timeSaved 1535089309830;
		getEmployee #mesquite.minimal.ManageTaxa.ManageTaxa;
		tell It;
			setID 0 1429675756028057823;
		endTell;
		getWindow;
		tell It;
			suppress;
			setResourcesState false false 147;
			setPopoutState 400;
			setExplanationSize 0;
```

```
		setAnnotationSize 0;
		setFontIncAnnot 0;
		setFontIncExp 0;
		setSize 1348 629;
		setLocation -8 0;
		setFont SanSerif;
		setFontSize 10;
		getToolPalette;
		tell It;
		endTell;
		desuppress;
	endTell;
	getEmployee  #mesquite.minimal.ManageTaxa.ManageTaxa;
	tell It;
		showTaxa #1429675756028057823 #mesquite.lists.TaxonList.TaxonList;
		tell It;
			setTaxa #1429675756028057823;
			getWindow;
			tell It;
```

```
newAssistant
#mesquite.lists.DefaultTaxaOrder.DefaultTaxaOrder;
newAssistant
#mesquite.lists.TaxonListCurrPartition.TaxonListCurrPartition;
setExplanationSize 30;
setAnnotationSize 20;
setFontIncAnnot 0;
setFontIncExp 0;
setSize 1201 562;
setLocation -8 0;
setFont SanSerif;
setFontSize 10;
getToolPalette;
tell It;
setTool
mesquite.lists.TaxonList.TaxonListWindow.ibeam;
endTell;
endTell;
showWindow;
getEmployee #mesquite.lists.ColorTaxon.ColorTaxon;
```

```
tell It;
    setColor Red;
    removeColor off;
endTell;
getEmployee
#mesquite.lists.TaxonListAnnotPanel.TaxonListAnnotPanel;
tell It;
    togglePanel off;
endTell;
endTell;
endTell;
getEmployee  #mesquite.trees.BasicTreeWindowCoord.BasicTreeWindowCoord;
tell It;
    makeTreeWindow #1429675756028057823
#mesquite.trees.BasicTreeWindowMaker.BasicTreeWindowMaker;
    tell It;
        suppressEPCResponse;
        setTreeSource  #mesquite.trees.StoredTrees.StoredTrees;
        tell It;
            setTreeBlock 1;
```

```
toggleUseWeights off;
endTell;
setAssignedID 870.1521201595448.1420569832980180438;
getTreeWindow;
tell It;
setExplanationSize 30;
setAnnotationSize 20;
setFontIncAnnot 0;
setFontIncExp 0;
setSize 1201 562;
setLocation -8 0;
setFont SanSerif;
setFontSize 10;
getToolPalette;
tell It;
setTool
mesquite.trees.BasicTreeWindowMaker.BasicTreeWindow.arrow;
endTell;
setActive;
```

```
getTreeDrawCoordinator
#mesquite.trees.BasicTreeDrawCoordinator.BasicTreeDrawCoordinator;
tell It;
    suppress;
    setTreeDrawer  #mesquite.trees.SquareTree.SquareTree;
    tell It;
        setNodeLocs
#mesquite.trees.NodeLocsStandard.NodeLocsStandard;
        tell It;
            branchLengthsToggle on;
            toggleScale on;
            toggleBroadScale off;
            toggleCenter on;
            toggleEven on;
        endTell;
        setEdgeWidth 2;
        orientUp;
        setCornerMode Right_Angle  50;
    endTell;
    setBackground White;
```

```
setBranchColor Black;
showNodeNumbers off;
showBranchColors on;
labelBranchLengths on;
centerBrLenLabels on;
showBrLensUnspecified on;
showBrLenLabelsOnTerminals on;
setBrLenLabelColor 250 10 10;
setNumBrLenDecimals 6;
desuppress;
getEmployee
#mesquite.trees.BasicDrawTaxonNames.BasicDrawTaxonNames;
tell It;
	setFontSize 12;
	setColor Black;
	toggleColorPartition on;
	toggleShadePartition off;
	toggleShowFootnotes on;
	toggleNodeLabels on;
	toggleCenterNodeNames off;
```

```
			toggleShowNames on;
			namesAngle ?;
		endTell;
	endTell;
		setTreeNumber 1;
		setDrawingSizeMode 0;
		toggleLegendFloat on;
		scale 0;
		toggleTextOnTree off;
		showWindow;
	endTell;
	desuppressEPCResponse;
	getEmployee #mesquite.trees.ColorBranches.ColorBranches;
	tell It;
		setColor Red;
		removeColor off;
	endTell;
	getEmployee #mesquite.ornamental.BranchNotes.BranchNotes;
	tell It;
```

```
setAlwaysOn off;
endTell;
getEmployee
#mesquite.ornamental.ColorTreeByPartition.ColorTreeByPartition;
tell It;
colorByPartition off;
endTell;
getEmployee
#mesquite.ornamental.DrawTreeAssocDoubles.DrawTreeAssocDoubles;
tell It;
setOn on;
setDigits 4;
writeAsPercentage off;
toggleCentred on;
toggleHorizontal on;
toggleWhiteEdges on;
setFontSize 10;
setOffset 0  0;
endTell;
```

```
                    getEmployee
#mesquite.ornamental.DrawTreeAssocStrings.DrawTreeAssocStrings;
                    tell It;
                        setOn on;
                        toggleCentred on;
                        toggleHorizontal on;
                        setFontSize 10;
                        setOffset 0  0;
                    endTell;
                    getEmployee #mesquite.trees.TreeInfoValues.TreeInfoValues;
                    tell It;
                        panelOpen false;
                    endTell;
                endTell;
            endTell;
            endTell;
end;
```

Supplementary material 6. Outputs of the regressions of log brain mass over log body corrected for phylogeny including (A) and excluding outliers (B).

Endocranial volume v body mass for 26 proboscidean specimens

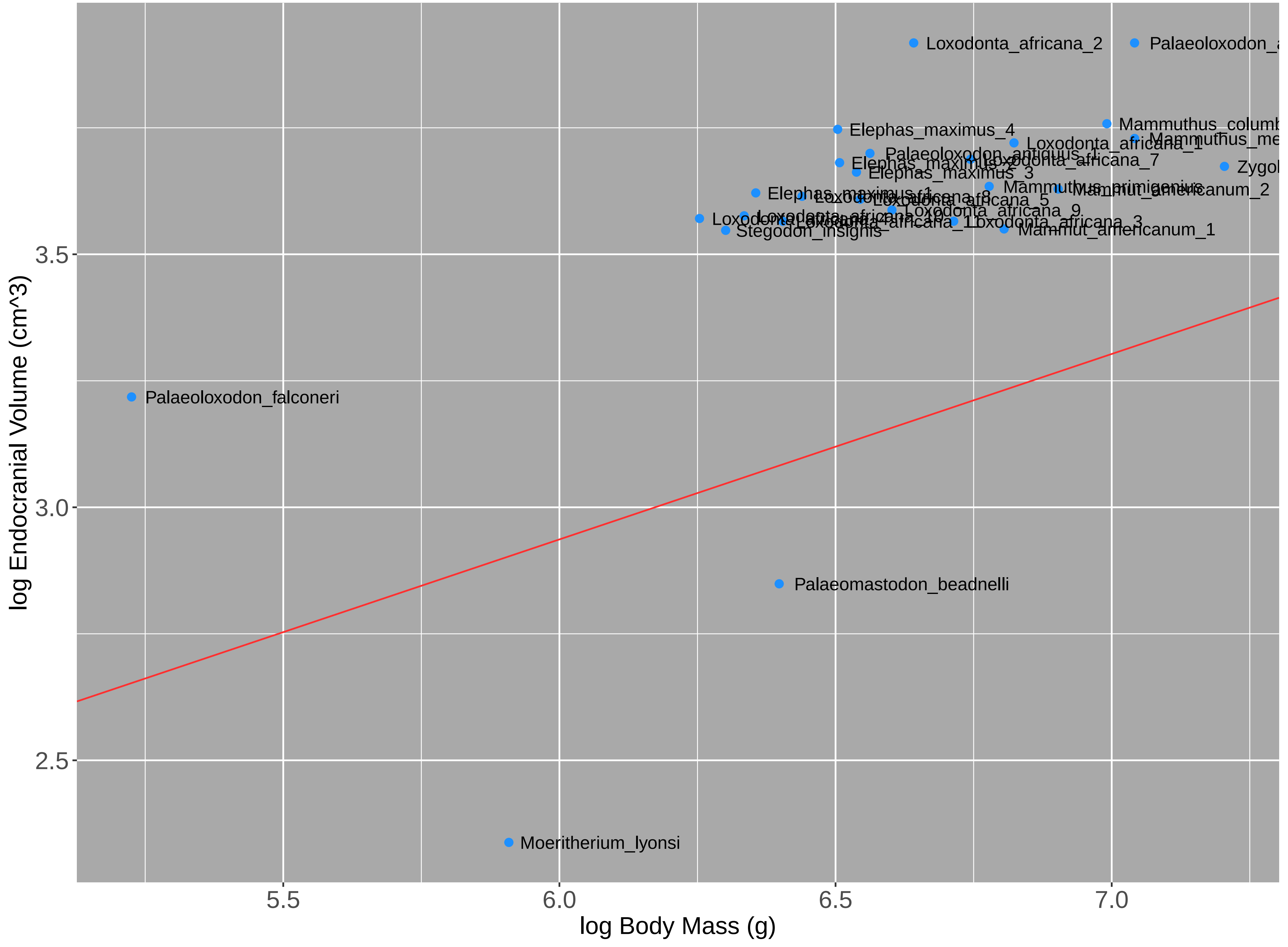

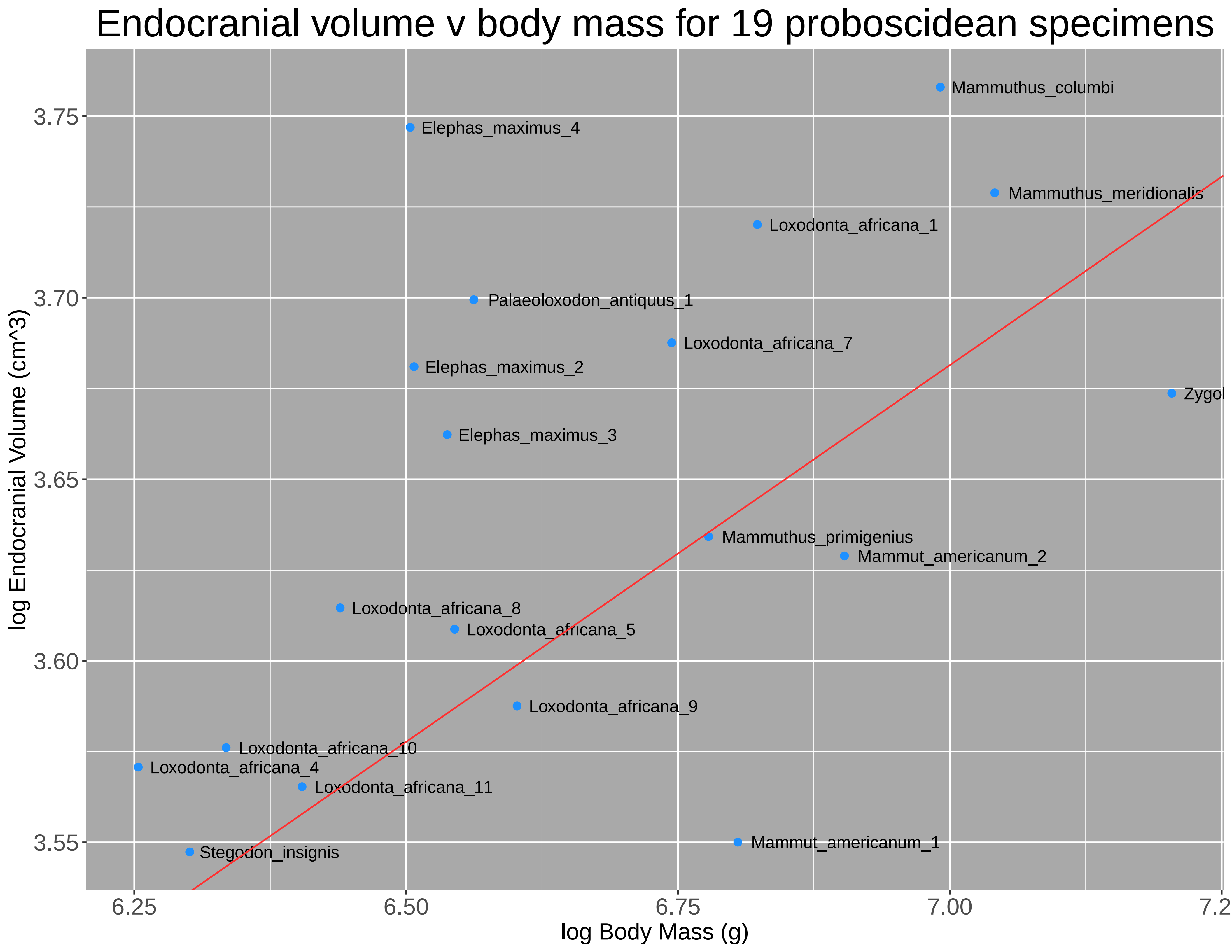
Endocranial volume v body mass for 19 proboscidean specimens
log Endocranial Volume (cm^3)
log Body Mass (g)
3.75
3.70
3.65
3.60
3.55
6.25
6.50
6.75
7.00
7.2
Mammuthus_columbi
Elephas_maximus_4
Mammuthus_meridionalis
Loxodonta_africana_1
Palaeoloxodon_antiquus_1
Loxodonta_africana_7
Elephas_maximus_2
Zygol
Elephas_maximus_3
Mammuthus_primigenius
Mammut_americanum_2
Loxodonta_africana_8
Loxodonta_africana_5
Loxodonta_africana_9
Loxodonta_africana_10
Loxodonta_africana_4
Loxodonta_africana_11
Mammut_americanum_1
Stegodon_insignis
